# Distinct Effects of Major Affective Disorder Diagnoses and Suicidal Symptom Severity on Inhibitory Control Function and Proinflammatory Cytokines: Single-Site Analysis of 800 Adolescents and Adults

**DOI:** 10.1093/ijnp/pyae043

**Published:** 2024-09-16

**Authors:** Ya-Mei Bai, Mu-Hong Chen, Ju-Wei Hsu, Hsiang-Hsuan Huang, Jia-Shyun Jeng, Shih-Jen Tsai

**Affiliations:** Institute of Brain Science, National Yang Ming Chiao Tung University, Taipei, Taiwan; Department of Psychiatry, College of Medicine, National Yang Ming Chiao Tung University, Taipei, Taiwan; Department of Psychiatry, Taipei Veterans General Hospital, Taipei, Taiwan; Institute of Brain Science, National Yang Ming Chiao Tung University, Taipei, Taiwan; Department of Psychiatry, College of Medicine, National Yang Ming Chiao Tung University, Taipei, Taiwan; Department of Psychiatry, Taipei Veterans General Hospital, Taipei, Taiwan; Department of Psychiatry, College of Medicine, National Yang Ming Chiao Tung University, Taipei, Taiwan; Department of Psychiatry, Taipei Veterans General Hospital, Taipei, Taiwan; Department of Psychiatry, College of Medicine, National Yang Ming Chiao Tung University, Taipei, Taiwan; Department of Psychiatry, Taipei Veterans General Hospital, Taipei, Taiwan; Department of Psychiatry, College of Medicine, National Yang Ming Chiao Tung University, Taipei, Taiwan; Department of Psychiatry, Taipei Veterans General Hospital, Taipei, Taiwan; Institute of Brain Science, National Yang Ming Chiao Tung University, Taipei, Taiwan; Department of Psychiatry, College of Medicine, National Yang Ming Chiao Tung University, Taipei, Taiwan; Department of Psychiatry, Taipei Veterans General Hospital, Taipei, Taiwan

**Keywords:** Suicide, inhibitory control, CRP, bipolar disorder, major depressive disorder

## Abstract

**Background:**

Inhibitory control function and proinflammatory cytokines play a role in the pathomechanisms underlying major affective disorders and suicidal behavior. However, the distinct or interactive effects of major affective disorders and suicidal symptom severity on inhibitory control function and proinflammatory cytokines remain unclear.

**Methods:**

This study included 287 patients with bipolar disorder, 344 with major depressive disorder, and 169 healthy controls. We categorized the participants into 3 groups based on Montgomery–Åsberg Depression Rating Scale (MADRS) item 10 (suicidal symptoms) score: 0, 2 or 3, and ≥4. The participants completed the go/no-go task and the measurements for C-reactive protein (CRP) and tumor necrosis factor-α (TNF-α) levels.

**Results:**

Errors in the go/no-go task were associated with suicidality (*P *= .040), regardless of the severity of suicidal symptoms and diagnosis. An elevated CRP level was especially associated with a Montgomery–Åsberg Depression Rating Scale item 10 score ≥4 (*P* = .001). An increased TNF-α level could distinguish bipolar disorder from major depressive disorder (*P* < .001).

**Discussion:**

Our study indicated the distinct effects of major affective disorder diagnosis and suicide symptom severity on inhibitory control function and CRP and TNF-α levels. Importantly, individuals with the poorest inhibitory control function and highest CRP levels had more severe suicidal symptoms.

**Significance Statement**

There were more errors on the go/no-go task and higher levels of C-reactive protein (CRP) in people with severe suicidal symptoms compared with people with less severe or no suicidal symptoms, regardless of the major affective disorder diagnoses (bipolar disorder and major depressive disorder). Patients with major affective disorders who make more mistakes on the go/no-go task and have higher CRP levels should have their suicide risk closely monitored.

## INTRODUCTION

Increasing evidence has shown that suicidal behavior and depression may be 2 distinct but highly comorbid and interactive psychiatric conditions (Orsolini et al., 2020; Jackson and Jabbi, 2022). According to the 2019 Global Burden of Disease Study, mental disorders continue to rank among the 10 leading causes of disease burden worldwide, with no evidence of this burden having decreased since 1990 (Collaborators, 2022). Among all mental disorders, depression was found to account for the largest proportion of disability-adjusted life-years in 2019 (37.3%), followed by schizophrenia (12.2%) and bipolar disorder (6.8%) (Collaborators, 2022). Alarmingly, although a suicide prevention program has been implemented in Taiwan since 2005, Taiwan has one of the highest suicide rates (>13/100 000) worldwide, as indicated by the World Health Organization (Centers for Disease Control and Prevention, 2012; Fazel, Wolf et al., 2013).

Studies have found that inhibitory control function, which refers to top-down inhibitory control of the prefrontal cortex, plays an important role in both major affective disorders (bipolar disorder and major depressive disorder) and suicidal behavior (Huang et al., 2022; Myers et al., 2023). In an at-risk-of-suicide clinical cohort of 136 veterans, including 95 with major depressive disorder and 12 with bipolar disorder, Myers et al. discovered that omission in the go/no-go task was associated with increased risk of actual suicide attempt during the 90-day follow-up and that errors were associated with other suicide-related events, such as interrupted or aborted attempts (Myers et al., 2023). The Scottish Family Health Study of Generation Scotland revealed that more severe depressive symptoms were associated with more errors and slower reactions in the go/no-go task (de Nooij et al., 2023). In a mild-to-moderate depression clinical cohort, Yitzhak et al. discovered that depressive symptoms were associated with less stable inhibitory performance in the go/no-go task (i.e., greater variability in the reaction time) (Yitzhak et al., 2023). Furthermore, accumulating evidence suggests a cross-diagnostic endophenotype of cognitive dysfunction between bipolar disorder and major depressive disorder and confirms greater cognitive function deficits, including inhibitory control dysfunction, in patients with bipolar disorder than in those with major depressive disorder (Cotrena et al., 2017; MacQueen and Memedovich, 2017; Ramirez-Martin et al., 2023). However, several studies have reported no difference in inhibitory control function between patients with 1 of the 2 major affective disorders vs patients with the other (Diler et al., 2014; Ryan et al., 2015). For example, Ryan et al. demonstrated that patients with bipolar disorder and those with major depressive disorder both performed worse (i.e., errors and reaction time) in the go/no-go task compared with a control group, with no significant differences between the performances of the 2 major affective disorder groups (Ryan et al., 2015). The distinct or interactive effects of major affective disorders and the effects of the severity of suicidal symptoms on inhibitory control function remain unknown.

Dysregulation of proinflammatory cytokines may be associated with the pathomechanisms of major affective disorders as well as suicidal behavior (Brundin et al., 2017; Miola et al., 2021; Aguglia et al., 2022). Miola et al. demonstrated that the C-reactive protein (CRP) level was significantly associated with suicidal behavior, regardless of a patient’s psychiatric diagnosis (affective or psychotic disorder) (Miola et al., 2021). The CRP level of individuals with high suicidal ideation or suicidal attempts was higher than that of nonsuicidal individuals (Miola et al., 2021). Aguglia et al. proposed CRP as a potential biomarker for high-lethality suicide attempts (Aguglia et al., 2022). Regarding other indicators, Janelidze et al. demonstrated that an increased level of tumor necrosis factor-α (TNF-α) may distinguish depressed individuals without suicidal symptoms from those who attempted suicide (Janelidze et al., 2011). A human postmortem brain study identified that TNF-α was upregulated in the prefrontal cortex of individuals who died by suicide, with this finding independent of the individuals’ psychiatric diagnosis (Wang et al., 2018).

Bipolar disorder is hypothesized to be a more severe systemic inflammatory disease than is major depressive disorder (Fernandes et al., 2016; Goldsmith et al., 2016). Goldsmith et al. reported that the TNF-α level was significantly elevated in patients with bipolar disorder (effect size: 0.43) and major depressive disorder (effect size: 0.35) compared with controls (Goldsmith et al., 2016). A meta-analysis of 2161 patients with bipolar disorder and 81 932 healthy controls found that the increase in CRP concentration was specific to bipolar disorder, regardless of symptom severity (Fernandes et al., 2016). However, the distinct or interactive effects of major affective disorders and the effects of the severity of suicidal symptoms on proinflammatory cytokines remain unknown.

This study aimed to clarify the common, distinct, or interactive effects of major affective disorders and the effects of the severity of suicidal symptoms on the performance of individuals in the go/no-go task and their proinflammatory cytokine profile, particularly their CRP and TNF-α levels. In addition, to find common factors (i.e., impulse control function and proinflammatory cytokines) related to suicidal symptoms, regardless of age, the present study enrolled individuals aged 12–64 years. We hypothesized that both major affective disorders and the severity of suicidal symptoms are associated with inhibitory control dysfunction, as measured using the go/no-go task, and elevated levels of CRP and TNF-α.

## METHODS

### Participants

In the present study, we recruited adolescents aged 12–17 years and adults aged 18–64 years with major affective disorders, namely bipolar disorder and major depressive disorder, who had no prior history of major physical diseases, such as epilepsy, autoimmune diseases, and neurovascular diseases, and no lifetime history of schizophrenia, organic mental disorder, or alcohol and substance use disorders. The Montgomery–Åsberg Depression Rating Scale (MADRS) was used for the assessment of overall depressive symptoms (Montgomery, 1980). Using the MADRS item 10 (suicidal symptoms) scores with the cut-off points of 2 and 4, we categorized the participants into 3 groups: 0 (enjoys life or takes it as it comes), 2 (weary of life; only fleeting suicidal thoughts) or 3, and ≥4 (feels better off dead; suicidal thoughts common and considered as possible solution but no specific plans or intention) (Montgomery, 1980; Ballard et al., 2015). Those who scored 1 in MADRS item 10 were excluded from the present study owing to the ambiguity of suicidal symptoms. We also enrolled healthy controls without any of the mentioned physical conditions or psychiatric disorders. All procedures contributing to this work complied with the ethical standards of the relevant national and institutional committees on human experimentation and with the Helsinki Declaration of 1975 as revised in 2008. All procedures involving human subjects or patients were approved by the institutional review boards of Taipei Veterans General Hospital with the approval numbers 2016-09-011C and 2020-02-016A. All participants and the parents of adolescent participants gave their written informed consent.

### Assessment of Inflammatory Makers

Using enzyme-linked immunosorbent assay kits (R&D Systems, Minneapolis, MN, USA), proinflammatory cytokines, including TNF-α and CRP, were measured in each participant. Fasting serum samples were collected in serum separator tubes and clotted for 30 minutes between 9:00 am and 12:00 pm. All samples were then stored at −80°C until use. All assays were performed according to the manufacturer’s instructions. Final absorbance of the mixture was measured and analyzed at 450 nm using an enzyme-linked immunosorbent assay plate reader with Bio-Tek Power Wave Xs and Bio-Tek’s KC junior software (Winooski, VT, USA). The standard range depended on the manufacturer’s instructions, and a linear regression, R^2^ value, of ≥0.95 represented a reliable standard curve.

### Measurement of Neurocognitive Functions

The go/no-go task was used for the measurement of inhibitory control function. Participants in the go/no-go task were instructed to react as soon as the × symbol appeared. When the + symbol appeared, they were not to press the key. They took the formal test to record their errors, omission, and reaction times (mean ± SD) after completing the pretest with all correct answers. The go/no-go task was commonly used in our previous studies (Chen et al., 2018; Bai et al., 2023).

### Statistical Analysis

For between-group comparisons, the F-test was used for continuous variables and Pearson test was used for categorical variables. Generalized linear models (GLMs) with adjustment of demographic data (age, sex, BMI, and education years) and nonsuicidal depressive symptoms (subtotal MADRS items 1−9 scores) were performed to examine the main effects of diagnoses (bipolar disorder vs major depressive disorder) and suicidal severity conditions (MADRS item 10 score: 0 vs 2 or 3 vs ≥4) and the interactive effect of both 2 conditions on the inhibitory control function. After adjusting for demographic data and nonsuicidal depressive symptoms, we further performed GLMs with gamma log link to assess the main effects of diagnoses and suicidal severity conditions, as well as the interactive effect of both conditions, on the proinflammatory cytokine levels. We further focused on the statistically significant effects of the GLMs for the further post-hoc analyses. Furthermore, we used the GLMs to assess associations between CRP levels and results of the go/no-go task among patients with prominent suicidal symptoms. Finally, we examined the effect of prior suicide attempts on inhibitory control function and proinflammatory cytokine levels among patients with prominent suicidal symptoms. A 2-tailed *P* value < .05 was considered statistically significant. All data processing and statistical analyses were performed using the SPSS version 17 software (SPSS Inc., Chicago, IL, USA).

### Data Availability

The data that support the findings of this study are available on request from the corresponding author. The data are not publicly available due to the ethical regulation in Taiwan.

## RESULTS

In all, 287 patients with bipolar disorder, 344 with major depressive disorder, and 169 healthy controls were included in the present study, with 492 having no suicidal symptom (MADRS item 10 score = 0), 190 scoring 2 or 3 in the MADRS item 10, and 118 having prominent suicidal symptoms (MADRS item 10 score ≥4) (Tables 1 and 2). Age did not differ between the 3 groups based on MADRS item 10 (*P* = .297), while age was younger (*P* < .001) in the healthy control group than in the other 2 diagnosis groups (Tables 1 and 2). Table 1 shows that patients scoring ≥4 in the MADRS item 10 had greater nonsuicidal depressive symptoms (*P* < .001), performed worse in the go/no-go task (all *P* < .05, except omission), and had elevated CRP levels compared with the control group. Patients with prominent suicidal symptoms had the highest rate (66.1%) of prior suicide attempts compared with the other 2 groups (*P* < .001) (Table 1). Table 2 shows that patients with major depressive disorder had greater suicidal (*P* < .001) and nonsuicidal depressive symptoms (*P* < .001) symptoms than did those with bipolar disorder and the control group. Patients with bipolar disorder had a higher rate of prior suicide attempts (40.1% vs 30.5, *P* = .015) than did those with major depressive disorder (Table 2). Patients in both disease groups performed worse in the go/no-go task (all *P* < .05) compared with the control group (Table 2). Patients with bipolar disorder had the highest TNF-α levels (*P* < .001) compared with the other 2 groups (Table 2).

**Table 1.** Demographic and Clinical Data, Stratified by Suicide Severity

Variables	Severity of suicidal symptoms based on MADRS item 10 scores (n = 800)			*P*	Post-hoc
	0 (A) (n = 492)	2 or 3 (B) (n = 190)	≥4 (C) (n = 118)		
Age (y, SD, n, %)	33.48 (14.97)	33.02 (15.37)	35.64 (15.12)	.297	
Adolescents	78 (15.9)	38 (20.0)	13 (11.0)	.110	
Adults	414 (84.1)	152 (80.0)	105 (89.0)		
Sex (n, %)				.001	
Male	183 (37.2)	50 (26.3)	27 (22.9)		
Female	309 (62.8)	140 (73.7)	91 (77.1)		
BMI (SD)	23.68 (4.74)	22.99 (4.25)	24.15 (5.31)	.085	
Education years (y, SD)	13.32 (3.52)	12.49 (3.20)	13.01 (3.08)	.016	B<A
Diagnosis (n, %)					
Bipolar disorder	191 (38.3)	71 (37.4)	25 (21.2)		
Major depressive disorder	132 (26.8)	119 (62.6)	93 (78.8)		
Control group (n, %)	169 (34.3)				
History of suicidal attempt (n, %)	79 (16.1)	64 (33.7)	78 (66.1)	<.001	C>B>A
MADRS scores (SD)					
MADRS item 10 scores (SD)	0.00 (0.00)	2.43 (0.50)	4.15 (0.36)	<.001	C>B>A
Subtotal MADRS items 1–9 scores (SD)	6.89 (8.03)	25.18 (6.80)	32.28 (5.77)	<.001	C>B>A
Go-no-go task (SD)					
Errors	0.61 (1.57)	1.19 (2.21)	1.35 (2.05)	<.001	C~B>A
Omission	0.30 (1.37)	0.90 (2.53)	0.58 (1.32)	<.001	B>A
Mean time (ms)	455.59 (95.17)	466.72 (93.00)	481.09 (102.28)	.026	C>A
s.d.	74.55 (34.49)	86.25 (34.61)	93.16 (44.61)	<.001	C~B>A
Proinflammatory cytokines (SD)					
CRP (ng/mL)	1557.24 (2211.40)	1742.40 (2485.85)	2604.00 (3729.69)	<.001	C>B~A
TNF-α (ng/mL)	960.84 (416.37)	908.13 (312.19)	912.65 (270.64)	.174	

Abbreviations: BMI, body mass index; CRP, C-reactive protein; MADRS, Montgomery–Åsberg Depression Rating Scale; TNF, tumor necrosis factor.

**Table 2.** Demographic and Clinical Data, Stratified by Diagnoses

Variables	BD (A) (n = 287)	MDD (B) (n = 344)	Control group (C) (n = 169)	*P*	Post-hoc
Age (y, SD, n, %)	36.25 (14.35)	36.08 (15.46)	24.47 (11.69)	<.001	A~B>C
Adolescents	21 (7.3)	39 (11.3)	69 (40.8)	<.001	
Adults	266 (92.7)	305 (88.7)	100 (59.2)		
Sex (n, %)					
Male	96 (33.4)	89 (25.9)	75 (44.4)	<.001	
Female	191 (66.6)	255 (74.1)	94 (55.6)		
BMI (SD)	24.78 (4.99)	23.32 (4.65)	22.09 (3.88)	<.001	A>B>C
Education years (y, SD)	13.41 (3.03)	13.02 (3.30)	12.63 (4.08)	.058	
History of suicidal attempt (n, %)	115 (40.1)	105 (30.5)		.015	
MADRS scores (SD)					
MADRS item 10 scores (SD)	0.95 (1.43)	1.97 (1.74)	0.00 (0.00)	<.001	B>A>C
Subtotal MADRS items 1–9 scores (SD)	13.23 (10.51)	23.63 (10.39)	0.35 (0.87)	<.001	B>A>C
Go-no-go task (SD)					
Error	1.09 (2.28)	0.94 (1.76)	0.28 (0.70)	<.001	A>C
Omission	0.50 (1.87)	0.63 (1.95)	0.17 (0.60)	.016	A~B>C
Mean time (ms)	473.50 (115.69)	465.62 (84.82)	435.08 (73.68)	<.001	A~B>C
SD	84.46 (46.18)	82.61 (32.95)	67.46 (20.40)	<.001	A~B>C
Proinflammatory cytokines (SD)					
CRP (ng/mL)	1912.43 (2458.50)	1944.93 (2850.55)	1103.96 (2032.49)	.001	A~B>C
TNF-α (ng/mL)	1081.75 (523.42)	881.70 (244.47)	823.69 (164.15)	<.001	A>B~C

Abbreviations: BD, bipolar disorder; BMI, body mass index; CRP, C-reactive protein; MADRS, Montgomery–Åsberg Depression Rating Scale; MDD, major depressive disorder; TNF, tumor necrosis factor.

GLM identified the main effects of the MADRS suicide item severity (0 vs 2−3 vs ≥ 4) on the errors (*P* = .042) in the go/no-go task and the CRP levels (*P* = .002) after adjusting for age, sex, BMI, diagnoses, education, and nonsuicidal depressive symptoms (Table 3). Furthermore, a history of suicide attempts was associated with higher errors in the go/no-go task (*P* = .004) but not CRP levels (*P* = .124) among patients with prominent suicidal symptoms. The main effects of the diagnosis condition (bipolar disorder vs major depressive disorder vs control group) were noted on the mean time (*P* = .045) and the SD of mean time (*P* = .013), as well as the TNF-α levels (*P* < .001) (Table 3). Surprisingly, we found no association between CRP levels and results of the go/no-go task among patients with prominent suicidal symptoms (all *P* > .05), with only a trend association (*P* = .075) between higher CRP levels and longer mean reaction time.

**Table 3.** Generalized Linear Models for the Go-no-go Task and Proinflammatory Cytokines^*a*^

Variables	df	Wald χ^2^	*P*	df	Wald χ^2^	*P*
	GNG: error			GNG: omission		
MADRS suicide item severity (S)	2	6.458	**.040**	2	4.507	.105
Diagnoses (D)	2	5.307	.070	2	0.055	.973
S*D	2	0.237	.888	2	0.005	.998
	GNG: mean time			GNG: SD of mean time		
MADRS suicide item severity (S)	2	3.017	.221	2	3.907	.142
Diagnoses (D)	2	6.317	**.042**	2	8.892	**.012**
S*D	2	4.913	.086	2	9.391	**.009**
	CRP			TNF-α		
MADRS suicide item severity (S)	2	12.837	**.002**	2	2.383	.304
Diagnoses (D)	2	3.306	.191	2	38.185	**< .001**
S*D	2	1.708	.462	2	5.316	.070

Abbreviations: BMI, body mass index; CRP, C-reactive protein; GNG, go/no-go task; MADRS, Montgomery–Åsberg Depression Rating Scale; TNF, tumor necrosis factor.

^*a*^Adjusting for age, sex, BMI, education years, and nonsuicidal depressive symptoms. Bold type indicates the statistical significance (*P* < .05).

**Figure 1.**
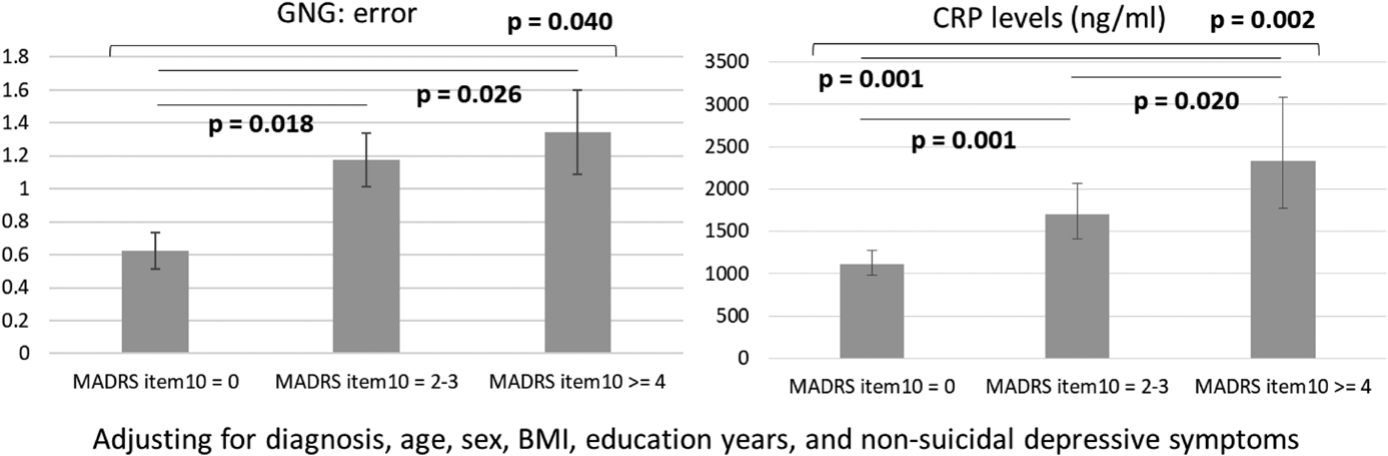
Generalized linear models for the inhibitory control function and proinflammatory cytokines between suicide severity groups. Abbreviations: BMI, body mass index; CRP, C-reactive protein; GNG, go/no-go task; MADRS, Montgomery–Åsberg Depression Rating Scale.

**Figure 2.**
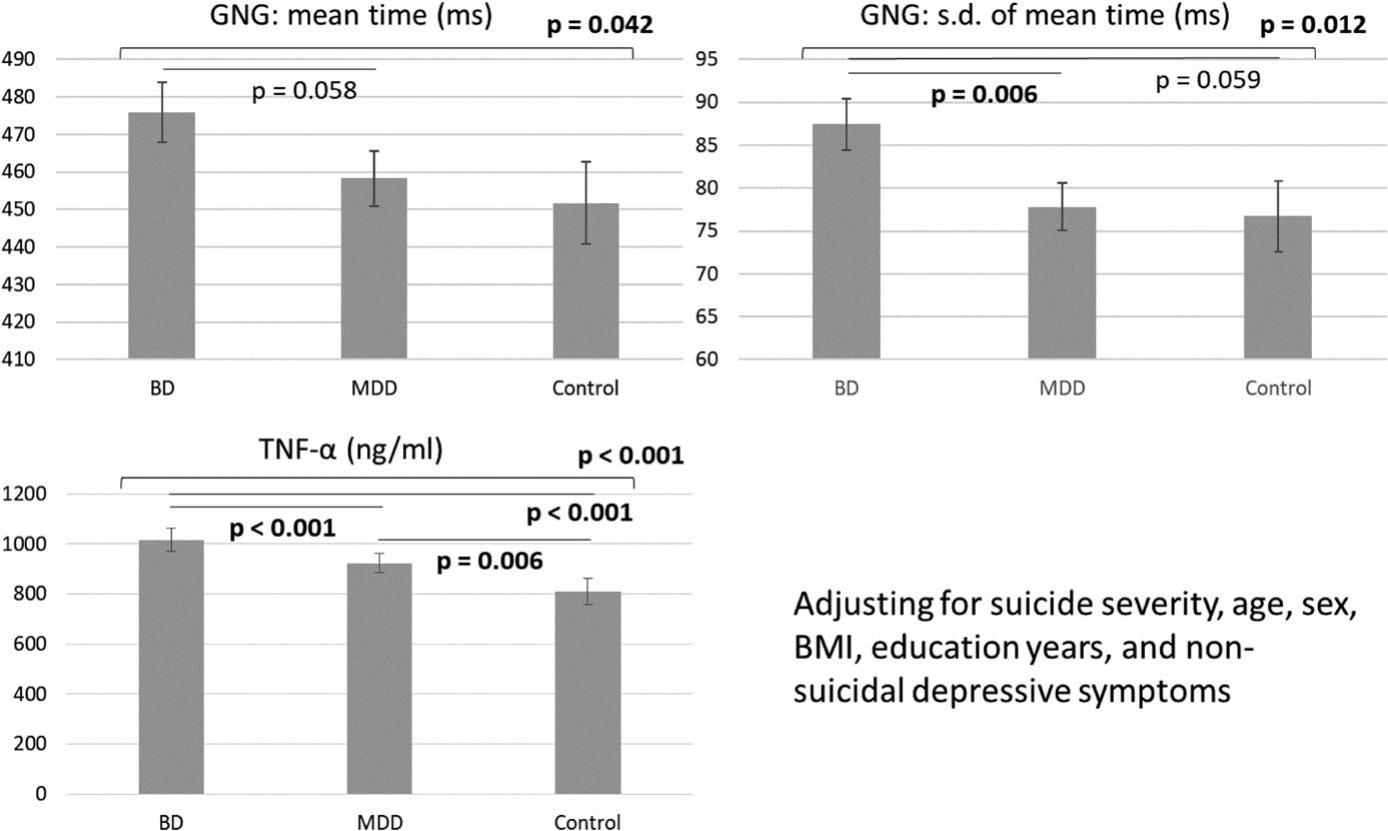
Generalized linear models for the inhibitory control function and proinflammatory cytokines between diagnoses. Abbreviations: BD, bipolar disorder; BMI, body mass index; GNG, go/no-go task; MDD, major depressive disorder; TNF, tumor necrosis factor.

Figures 1 and 2 showed post-hoc analyses of the effects of the MADRS suicide item severity and diagnosis condition on the inhibitory control function and proinflammatory cytokine levels. Specifically, the errors in the go/no-go task did not differ between those scoring 2 or 3 and ≥4 in the MADRS item 10 but were significantly higher in those 2 groups (*P* = .026, .018, respectively) than in those scoring 0 in the MADRS item 10 (Figure 1). Patients with prominent suicidal thoughts (*P* = .001) had the highest levels of CRP, followed by those who scored 2 or 3 on MADRS item 10 (*P* = .001), compared with those who did not have suicidal thoughts (Figure 1). As shown in Figure 2, the TNF-α levels were highest (*P* < .001) in the patients with bipolar disorder, followed by those with major depressive disorder and the control group. Patients with bipolar disorder had a higher SD of mean time (*P* = .006) than those with major depressive disorder (Figure 2).

## DISCUSSION

We report some crucial clinical findings from a large cohort of 800 adolescent and adult individuals. First, inhibitory control dysfunction, particularly errors in the go/no-go task, was associated with suicidality, regardless of the severity of suicidal symptoms (MADRS item 10 score: 2 or 3 vs ≥4), nonsuicidal depressive symptoms, and diagnosis of major affective disorder. Second, CRP level was more strongly associated with severe suicidal symptoms (MADRS item 10 score ≥4) than with mild suicidal symptoms or major affective disorder diagnosis. Third, regardless of symptom severity, bipolar disorder can be distinguished from major depressive disorder based on an elevated TNF-α level and less stable inhibitory control, as was evidenced by the SD of the reaction time in the go/no-go task.

Errors in the go/no-go task and CRP level may serve as biomarkers of suicidal symptoms and highly severe suicidal symptoms, respectively (Miola et al., 2021; Aguglia et al., 2022). Among patients with prominent suicidal symptoms, prior suicide attempt history was associated with even higher errors in the go/no-go task but not CRP level. In the go/no-go task, errors, also called false alarms, occur when the button is pressed by mistake. They are widely accepted as indicators of inhibitory control dysfunction and motor impulsivity (Meule, 2017). Venables et al. and Buchman-Schmitt et al. suggested that the inhibitory control/disinhibition trait plays a crucial role in the pathomechanism of suicidal spectrum symptoms, including thoughts, plans, and attempts (Buchman-Schmitt et al., 2017; Venables et al., 2018). A study on 444 identical and fraternal twins discovered that the disinhibition trait was considerably heritable, with an estimated additive genetic influence of 0.45, and was associated with suicidality (thoughts, plans, and attempts) (Venables et al., 2018). Our previous population-based cohort study determined that the incidence of suicide coaggregated within families, independent of psychiatric comorbidities (Tsai et al., 2023). These results align with the finding of the present study—that inhibitory control dysfunction may be a trait biomarker of suicidality, independent of suicidal symptom severity, nonsuicidal depressive symptoms, and diagnoses of major affective disorders. Additionally, we found that individuals with a MADRS item 10 score ≥4 exhibited the highest levels of CRP, followed by people with less severe suicidal thoughts, compared with individuals in the nonsuicidal groups, which may imply that CRP is a state and trait biomarker of suicidal symptoms. Miola et al. also discovered an association between an elevated CRP level and high suicidal ideation, regardless of diagnoses (Miola et al., 2021). Yan et al. claimed that inflammation sounds the alarm for suicide risk (Yan et al., 2021). We posit that this association may be especially true in high-risk individuals, such as those with strong disinhibition.

Interestingly, we found that both poor impulse control and elevated levels of CRP were associated with the prominent suicidal symptoms among patients with major affective disorder. However, we found no correlation between the 2 conditions, which may partially suggest independent effects of impulse control function and proinflammatory cytokines on suicidality pathophysiology (Loas et al., 2016). Loas et al. examined associations between impulsivity, suicidal symptoms, and CRP levels among 122 inpatients with major affective disorders and demonstrated that patients with suicidal symptoms exhibited higher Barratt impulsivity scale scores and CRP levels compared with those without (Loas et al., 2016). They also found no association between total Barratt impulsivity scale scores and CRP levels (Loas et al., 2016). A study of youths with major depressive disorder and prior suicidal attempt history indicated no association between CRP levels and anger control-out, an index of impulsivity (Peng et al., 2024). Findings of Loas et al. and Peng et al. were consistent with our findings. Coryell et al. further supported that higher levels of impulsivity and dysregulated cytokine levels were associated with suicidal symptoms but stated that cytokine levels failed to explain the relationship between impulsivity and suicide attempt history (Coryell et al., 2018). The complex relationship between the impulse control function, CRP levels, and suicidal symptoms requires further in-depth investigation.

Elevated TNF-α level can potentially distinguish bipolar disorder from major depressive disorder (Goldsmith et al., 2016; Poletti et al., 2021). Goldsmith et al. reported that patients with bipolar disorder (effect size: 0.43) and major depressive disorder (effect size: 0.35) had a substantially higher TNF-α level than did the controls (Goldsmith et al., 2016). According to a machine learning study on 54 cytokines, chemokines, and growth factors, bipolar disorder diagnosis was predicted by high levels of inflammatory markers such as C-C motif chemokine ligand 3, interleukin-9 (IL-9), and TNF-α, whereas major depressive disorder diagnosis was predicted by high levels of proinflammatory markers such as IL-1β and IL-6 (Poletti et al., 2021). Our findings also indicate that TNF-α level could distinguish the 2 major affective disorder groups from the control group and further distinguish between the 2 disorders themselves. Furthermore, in our study, the SD of the reaction time in the go/no-go task was greater in the bipolar disorder group than in the major depressive disorder group, which may reflect more instability of inhibitory control in bipolar disorder than in major depressive disorder (Cotrena et al., 2016; Fortgang et al., 2016). Cotrena et al. revealed that patients with bipolar disorder performed worse than patients with major depressive disorder on measures of sustained attention and inhibitory control (Cotrena et al., 2016).

Our study has some limitations. First, we assessed inhibitory control function only by evaluating performance in the go/no-go task and by measuring the levels of specific proinflammatory cytokines, namely CRP and TNF-α. Additional studies are required to evaluate the associations of different aspects of cognitive function and other proinflammatory cytokines with suicidal behavior in the 2 major affective disorders. Second, the patients continued their psychotropic medications throughout our cognitive evaluation and cytokine measurement so that their affective and suicidal symptoms were not exacerbated, which was the more ethical choice. The medication details were not available in our study. Nevertheless, further studies with a drug-free design would be necessary to validate our findings. Third, the major affective disorder diagnoses were given by the senior psychiatrists Y.M.B. or M.H.C., respectively, using a clinical diagnostic interview based on the criteria of the Diagnostic and Statistical Manual of Mental Disorders, Fifth Edition. The well-experienced clinical practice ensured that major affective disorder diagnoses were diagnostically valid, although the formal diagnostic interview manuals, such as Structured Clinical Interview for DSM Disorders, were not used in the present study. Fourth, we enrolled 129 adolescents and 671 adults in the present study. Because of the relatively small sample size of adolescents, separate analyses by age were limited. As mentioned, our study goal was to discover common factors related to suicidal symptoms, regardless of age and diagnosis. However, further studies with a large sample size of adolescents would be required to validate our findings.

In conclusion, inhibitory control dysfunction was associated with suicidal behavior, regardless of suicidal symptom severity, nonsuicidal depressive symptoms, and diagnoses. An elevated CRP level was associated with more severe suicidal symptoms. Patients with bipolar disorder were more likely to have an increased TNF-α level and perform worse in the go/no-go task compared with those with major depressive disorder. The neuromechanisms underlying the distinct effects of the 2 major affective disorders and the effects of suicidal symptom severity on cognitive function and proinflammatory cytokines require further investigation.

## Data Availability

The data that support the findings of this study are available on request from the corresponding author. The data are not publicly available due to the ethical regulation in Taiwan.

## Notes

The study was supported by grant from Taipei Veterans General Hospital (V111C-010, V111C-040, V111C-029, V112C-033, V113C-010, V113C-011, V113C-039), Yen Tjing Ling Medical Foundation (CI-109-21, CI-109-22, CI-110-30, CI-113-30, CI-113-31, CI-113-32), Ministry of Science and Technology, Taiwan (MOST110-2314-B-075-026, MOST110-2314-B-075-024 -MY3, MOST 109-2314-B-010-050-MY3, MOST111-2314-B-075 -014 -MY2, MOST 111-2314-B-075 -013, NSTC111-2314-B-A49-089-MY2), Taipei, Taichung, Kaohsiung Veterans General Hospital, Tri-Service General Hospital, Academia Sinica Joint Research Program (VTA112-V1-6-1, VTA113-V1-5-1), and Veterans General Hospitals and University System of Taiwan Joint Research Program (VGHUST112-G1-8-1, VGHUST113-G1-8-1). The funding source had no role in any process of our study.

## Interest Statement

All authors declared no conflict of interest.
